# Editorial: Dioecy in Fruit Crops: The Gender Rise and Decline and Its Agronomic Impact

**DOI:** 10.3389/fpls.2021.719588

**Published:** 2021-07-15

**Authors:** Raffaele Testolin, Sarah M. Pilkington, Takashi Akagi

**Affiliations:** ^1^Department of Agricultural, Food, Animal and Environmental Sciences, University of Udine, Udine, Italy; ^2^Plant & Food Research, The New Zealand Institute for Plant and Food Research Ltd., Auckland, New Zealand; ^3^Graduate School of Environmental and Life Science, Okayama University, Okayama, Japan

**Keywords:** dioecious crops, sex determination, sex chromosomes, dioecy evolution, sex transition

Sexuality in animals is a rather stable trait, with the male genetic determinant operating in a female background, but is more complex in flowering plants.

Hermaphroditism (perfect flowers with both sexes expressed) is recognized as being the most ancient state in the evolution of Angiosperms. It has evolved into monoecism (separated sexes in the same individual) and gymnodioecism (individuals carrying only female flowers), which in turn has evolved to dioecism (male and female sexes in different individuals). These transitions could have occurred many times, with reversion to hermaphroditism occurring frequently as well.

This complex and unstable sexuality of plants has attracted the interest of scientists since 1,715 when Sébastien Vaillant, botanist at the “Jardin Royal des Plantes médicinales” (now “Le Jardin des Plantes”) in Paris, hypothesized the occurrence of sexual dimorphism in pistachio (*Pistacia vera*). Dioecy occurs in 5–6% of flowering plants but could reach 20% among the crop species and is therefore of economic interest, prompting this Frontiers Research Topic.

The Research Topic was launched in July 2019 and collected 115 potential contributors, most of which became irresponsive after the spread of Sars-Cov-2 pandemic. However, we are happy, as editors, to have collected two excellent reviews and original papers on persimmon (*Diospyros*) and date palm (*Phoenix*).

In short, Montalvão et al. offer an exhaustive view of the diversity and dynamics of sex determination in plants. The authors describe in their report that dioecy was found in some 15,600 species, spread over 175 families and 987 genera and suggest that the evolution took place independently hundreds, if not thousands, of times. With the control of gender being rather unstable, the reversion from dioecy to hermaphroditism might have occurred frequently as well. Indeed, two mutations or even a single mutation could drive the reversion.

The key message that emerges from this well-documented review is that while the formal genetic model—the “two-genes model”—first postulated by Mogens Westergard in 1958 and better shaped by Brian and Deborah Charlesworth later on, remains substantially true, the molecular investigation of the recent years would suggest that the genes involved are not always the same. The scientists list 25 different genes involved in dioecy, either to suppress femaleness or to promote maleness. What is clearly emerging is that different genes are involved in the same pathway and produce analogous phenotypic mutations. This is the case, for instance, for the nine genes involved in the cytokinin signaling, a key plant hormone, the level of which determines the gynoecium formation. Similarly, five different genes are involved in the micro-sporogenesis process that occurs in the tapetum and leads to the release of fertile pollen in male individuals.

Suppressors and promoters are connected either through a genetic linkage in the sex-determining region (SDR), where recombination between the sex-determinants is suppressed, or through an epistatic genetic interaction, that would not require linkage.

Indeed, dioecy might have developed from hermaphroditism through the intermediate step of gynodioeciousness (female individuals, where the pollen development is arrested) or through monoecy. The evolution of dioecy from monoecy would require a single gene that determines the sexual orientation, independent of the number of genes that control the development of stamens and carpels.

This aspect of dioecy has been revised for this Research Topic by Cronk and Müller, who analyzed species like *Diospyros, Populus*, and *Salix*. The authors point out that the separation of unisexual flowers has already occurred in the monoecious ancestor; therefore, the evolution toward dioecy may be a simple matter of separating pre-existing epistatic regulatory architecture to different individuals, which may be accomplished by a single master regulator gene (the ARR17 gene for *Populus* and the MeGI gene for *Diospyros*), either in a hemizygous state or toggled on–off by a suppressor (YARR17-IR and OGI, respectively, for *Populus* and *Diospyros*).

The Research Topic includes two valuable papers on persimmon and date palm. The first of two was preceded in the literature by several papers of the same authors on the complex, and in some instances incomplete, dioecy of this crop; while the second one introduces for the first time a molecular analysis of the SDR in *Phoenix*.

The contribution from Masuda et al. provides a deep insight into *Diospyros*, a genus that contains around 450 different species all dioecious or sub-dioecious and is becoming a model for the study of the fine tuning of sex expression. The authors focused on a transition out of dioecy into monoecy in hexaploid *Diospyros kaki* (Oriental persimmon), which is based on their previous study for epigenetic silencing of the sex determinants, OGI and MeGI. They studied a cultivar with high-frequent male flowers, uncovering the mechanism by which the epigenetic silencing of MeGI/OGI is regulated. The male-rich cultivar showed strong DNA methylation in the OGI promoter, as well as in other female-rich cultivars, while OGI expression in the male-rich cultivar is released from silencing. Further transcriptomic approaches suggested gene networks relating stress- or jasmonate signal might involve the release of OGI silencing *via* chromatin remodeling or histone-methylations. Masuda et al. provided an insight into the molecular mechanism to generate a well-balanced monoecious system evolved from dioecy.

The last paper included in this Research Topic on dioecy is from Torres et al. who reported updated information on date palm (*Phoenix dactylifera*), the most recent species on which the gender control has been explored at the molecular level. In date palm, there is the classical X/Y chromosome system, but the evolution of the Y chromosome, in absence of recombination, has led to a large-scale re-arrangement of the sex-determining region, detected by the analysis of the density of k-mers in the assembled scaffolds of the SDR region sequenced in three different male genotypes. The degeneration of the non-recombining Y-region is known among the evolutionists as the “Muller's ratchet”, according to which the lack of recombination (the “ratchet”) forces the degeneration of the region. Torres et al. provide experimental evidence of this evolutionary mechanism.

## Author Contributions

All authors listed have made a substantial, direct and intellectual contribution to the work, and approved it for publication.

## Conflict of Interest

The authors declare that the research was conducted in the absence of any commercial or financial relationships that could be construed as a potential conflict of interest.

